# On enhancing the noise-reduction performance of the acoustic lined duct utilizing the phase-modulating metasurface

**DOI:** 10.1038/s41598-023-49592-2

**Published:** 2023-12-13

**Authors:** Yang Ou, Yonghui Zhao

**Affiliations:** State Key Laboratory of Mechanics and Control for Aerospace Structures, Nanjing University of Aeronautics and Astronautics, No. 29 Yudao Street, Nanjing, 210016 China

**Keywords:** Applied physics, Acoustics

## Abstract

This work proposes a noise-reduction structure that integrates phase-modulating metasurface (PMM) with acoustic liners (ALs) to enhance the narrow band absorption performance of a duct with relatively small length-diameter ratio. The PMM manipulates the wavefront by introducing different transmission phase shifts based on an array of Helmholtz resonators, so that the spinning wave within the duct can be generated. Compared with the plane wave, the generated spinning wave has a lower group velocity, which results in a greater traveling distance over the ALs in the duct. The optimization design is performed to determine the final structural parameters of the PMM, which is based on the predictions of the amplitude and phase shift of the acoustic wave at the outlet of the PMM using the theory of passive phased array. With the manipulation of the PMM, the incident plane wave is modulated into a spinning wave, and then enters into the acoustic liner duct (ALD), whose structural parameters are optimized by maximizing the transmission loss using the mode-matching technique. Finally, the noise-reduction performance of this combined structure is evaluated by numerical simulations in the presence of grazing flow. The results demonstrate that, compared with the traditional ALD, the proposed structure exhibits a significant increase in transmission loss within the considered frequency band, especially near the peak frequency of the narrow band noise.

## Introduction

Reduction of noise generated due to ground or air traffic is currently and will remain an important topic in the future. One of the most effective ways to solve this problem is to use the acoustic absorbers. Absorbers placed on the surface of a structure is usually called acoustic liners (ALs), which is widely employed in aircraft gas turbine engine noise-reduction^1^. Traditionally, ALs are typically fabricated by a hard-backed honeycomb and micro-perforated plate (MPP), called single-degree-of-freedom (SDOF) liners^2,3^. However, the traditional SDOF liners produce a narrow absorption spectra and the maximum absorption occurs at the resonant frequency which is dependent on the depth of honeycomb core. To achieve the aim of noise-reduction over a broad range of frequencies, a septum can be used to separate honeycomb into two parts, forming two-degree-of-freedom (2DOF) liners^4,5^. 2DOF liners are capable to cover the necessary source spectrum. However, due to construction difficulties and larger thickness, 2DOF liners are seldom used in aircraft engineering.

In recent years, further enhancement of acoustic absorbing performance of a lined duct in a limited length has received widespread attention. To this end, the non-uniform AL structure, characterized by the spatial variations of the impedance, has been proposed. Many theoretical analysis methods of the duct lined with circumferential^6–8^ and axial non-uniform^9,10^ absorbers have been developed. In this regard, Watson evaluated the acoustic absorbing performance of circumferentially segmented duct, and concluded that the circumferentially segmented absorption structure yields better broadband performance than the uniform absorption structure^11^. Palani et al. developed a novel non-uniform acoustic metasurface that incorporates a slanted porous septum design with varying open areas and a multiple folded cavity metasurface concept to enhance broadband absorption^12^. Jiang et al. developed an axially symmetrical flow duct with an azimuthally non-uniform AL on the duct wall to improve the absorption efficiency of spinning wave^13^.

As is well known, the noise-reduction performance of a lined duct is closely related to its geometric dimensions. Suppose a cylindrical duct with length $L$ and diameter $D$ is mounted with ALs on its inner wall, then the transmission loss of the duct is proportional to the length-diameter ratio^14^, that is ${\text{TL}} \propto L/D$. Therefore, it is difficult to achieve good noise-reduction performance for the duct with relatively small length-diameter ratio. Fortunately, wave manipulation technique based on metasurface may be a promising remedy to this issue. Wave manipulation using artificial materials is a hot topic in the field of materials physics. Introducing the concept of metasurfaces to the fields of materials science and physics via the generalized Snell's law has created opportunities for manipulating optical waves and led to many new applications^15,16^. Based on these pioneering works in optics, significant advancements have also been achieved in acoustics, including the utilization of acoustic metasurfaces for wave manipulation. Acoustic metasurfaces, as a type of wavefront manipulation devices, possess the capabilities to achieve anomalous reflection, anomalous refraction, focusing and absorption of acoustic waves^17–20^. Recently, there has been a significant surge of interest in the field of acoustic wave manipulation using PMM that is usually composed of a set of Helmholtz resonators. This kind of PMM is widely utilized to manipulate wavefront by adjusting its geometrical dimensions^21–23^. Li et al. have developed a comprehensive theory for analyzing transmission and reflection properties of a metascreen consisting of four Helmholtz resonators in series with a straight duct^24^. In this study, the refracted acoustic field was controlled by selecting an appropriate phase profile. Xia et al. utilized a two-layer Helmholtz resonator structure to implement an acoustic focusing lens^25^. Sun et al. utilized metagratings, which are complex elements composed of three Helmholtz resonators, to manipulate wavefront orientation by combining traditional diffraction and interference with the free phase modulation capability of the local resonant structures^26^. Ismail et al. presented a study on transmission loss through an acoustic metasurface based on Helmholtz resonators^27^. To achieve the expected noise-reduction performance, they performed a parametric study on the sensitivity of design variables, including the number of cells, thickness of metasurface and multilayering. Tang et al. achieved an asymmetric accelerating beam both numerically and experimentally by utilizing a bilayer binary acoustic metasurface consisting of a rectangular cavity (bit ‘0’) and a waveguide with seven Helmholtz resonators (bit ‘1’)^28^. Based on the conversion of angular momentum of acoustic orbits, Liu et al. proposed the concept of acoustic geometric phase element arrays. Well-defined geometric phases can be obtained through a variety of topological charge conversion processes, which provided a new approach to acoustic wave control^29^.

This paper focuses on the design of a noise-reduction structure to absorb the narrowband exhaust noise in a duct with relatively small length-diameter ratio. The spectrum of narrowband noise typically has a high peak representing the main harmonic component. Narrowband noise usually occurs in the scenes of the car exhaust pipe^30^, the engine cooling fan^31^, the water pipe^32^, etc. Narrowband noise usually exhibits a whistle-like sound. Kanai^30^ reported a narrowband noise with a peak frequency of 3800 Hz in the exhaust duct, which is the target frequency of the noise-reduction structure in this paper. As mentioned above, achieving good acoustic absorption becomes more challenging when dealing with the duct with a relatively small length-diameter ratio. In this case, it may not be feasible to use the ALs alone in the duct. Based on the manipulation capabilities of acoustic waves of metasurface, this paper proposed a combined noise-reduction scheme to create an efficient acoustic absorption for a duct with a relatively small length-diameter ratio. To be specific, the new noise-reduction structure consists of PMM and ALD in series. The PMM is meticulously designed to generate a gradient phase distribution, which transforms the plane wave into a desired spinning wave, and then efficiently absorbed by the ALs. Compared with the plane wave, the generated spinning wave in higher-order circumferential mode exhibits a lower group velocity along the axis of the duct, which yields greater travelling distance in the lined duct^33^. It makes sense, therefore, that a better noise-reduction performance can be achieved.

## Overall design of the noise-reduction structure

**Figure 1:**
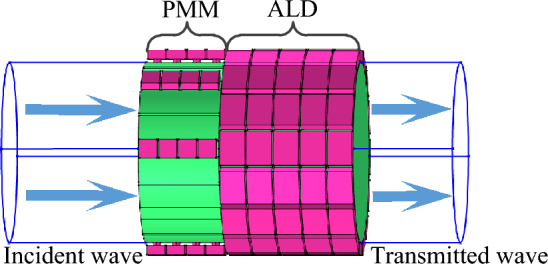
The proposed PMM-ALD noise-reduction structure.

The basic idea of enhancing the noise-reduction performance of a lined duct is to utilize a well-designed metasurface to manipulate the phase of plane wave such that a spinning wave in the higher-order mode is produced and then enters into a lined duct to achieve a more efficient acoustic absorption. In this way, the noise-reduction structure proposed in this paper can be divided into two parts, namely, PMM and ALD, they are shown in Fig. 1.

**Figure 2:**
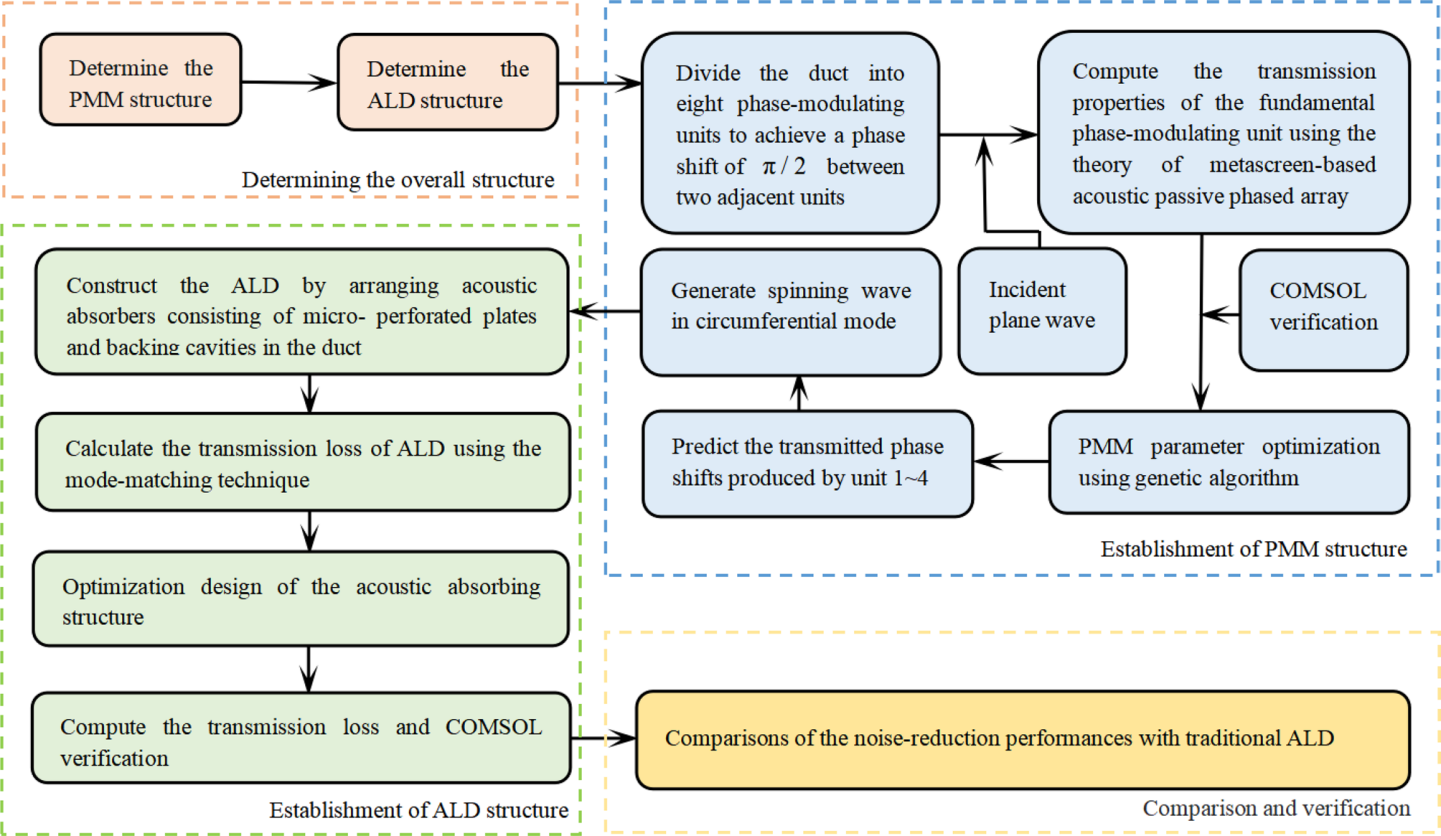
Flowchart of the overall design process of the PMM-ALD structure.

The PMM structure uses a set of Helmholtz resonators in series to manipulate the phase of the transmitted wave. In our design, the PMM is arranged on the inner wall of the duct, shifting the phase of the acoustic wave travelled in each passage to generate a spinning wave at the PMM exit. Compared with the plane wave, the generated spinning wave in higher-order circumferential mode exhibits a lower group velocity. As a result, the contact time between the spinning wave and the ALD is prolonged, thus a greater transmission loss is expected. To attenuate the generated spinning wave transmitted from the PMM exit, the ALs mounted on the inner wall of the duct is constructed with micro-perforated plates and backing cavities. The ALs, together with the duct, is called the ALD structure. The flowchart of the overall design process, including theoretical calculation methods and parameter optimization, is depicted in Fig. 2.

## Fundamental phase-modulating unit and transmission properties

**Figure 3:**
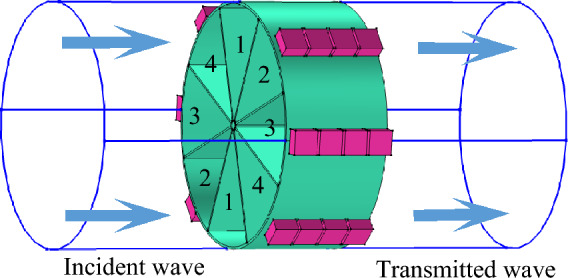
Spinning wave generation using eight phase-modulating units.

To obtain a circumferential acoustic mode, the complete phase coverage (that is an integer multiple of ${2}\pi$) is necessary. To this end, the PMM structure is designed to partition the cross-section of the duct into eight phase-modulating units, as shown in Fig. 3. In each unit, a total of four Helmholtz resonators in series are used for phase-modulating purpose. This design means that the phase difference between two adjacent units is $\pi {/2}$. In this case, the entire PMM structure is capable of generating a phase difference of ${4}\pi$ so that the spinning wave in a circumferential mode of 2 is generated. In Fig. 3, unit 1 employs a null structure, while units 2–4 are designed with different structural parameters to achieve distinct phase shifts.

**Figure 4:**
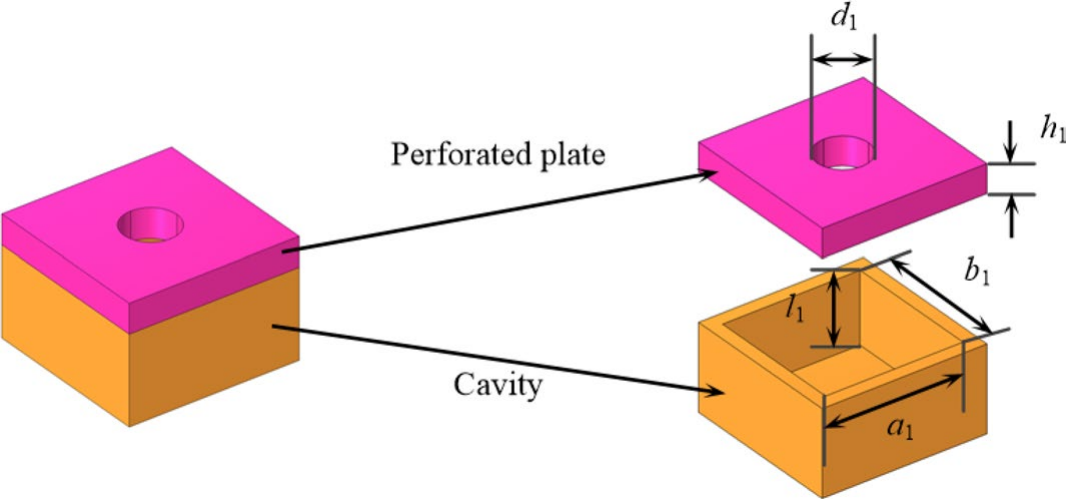
The fundamental resonance element (Helmholtz resonator).

**Figure 5:**
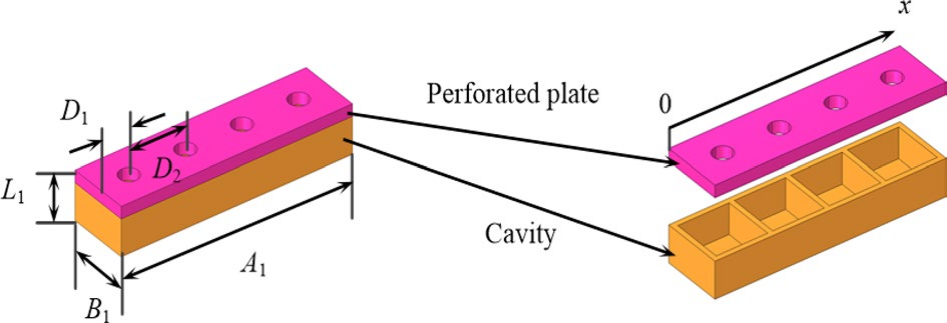
The fundamental phase-modulating metasurface.

**Figure 6:**
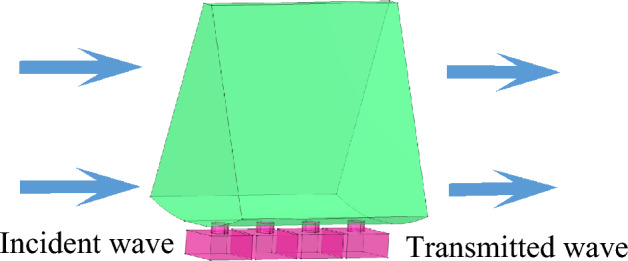
The fundamental phase-modulating unit of PMM.

Helmholtz resonator is used as the fundamental resonance element in unit 2–4, which comprises a perforated plate and a backing cavity, as illustrated in Fig. 4. The diameter and height of the hole on the perforated plate is denoted by $d_{1}$ and $h_{1}$, respectively. The length, width and height of the backing cavity are $a_{1}$, $b_{1}$ and $l_{1}$, respectively. Each fundamental phase-modulating metasurface includes four resonance elements, as illustrated in Fig. 5. Integrating the fundamental phase-modulating metasurface with a 1/8 sector duct yields the fundamental phase-modulating unit, as shown in Fig. 6.

The impedance transfer method^34^ is used to establish a set of theoretical prediction formula for the transmission characteristics of the fundamental phase-modulating unit. First, the acoustic impedance of the hole and the cavity can be written as

1 $$ Z_{h} = \frac{{\rho_{0} c_{0} }}{{S_{h} }},Z_{c} = - {\text{i}}\frac{{\rho_{0} c_{0} }}{{a_{1} b_{1} }}\cot \left( {k_{x} l_{1} } \right) $$

where, $S_{h} = \pi \left( {d_{1} /2} \right)^{2}$ is the sectional area of the hole, $\rho_{0}$, $c_{0}$ are the density of air and the acoustic velocity in air, respectively. In the absence of grazing flow, the axial component of wave number $k_{x} = k$, $k$ is the wave number. In the presence of grazing flow with Mach number $M_{x}$, $k_{x} = k/\left( {1 + M_{x} } \right)$.

According to the impedance transfer method, the acoustic impedance in duct above the cavity can be expressed as

2 $$ Z_{d} = Z_{h} \frac{{Z_{c} + {\text{i}}Z_{h} \tan \left( {k_{x} h_{a} } \right)}}{{Z_{h} + {\text{i}}Z_{c} \tan \left( {k_{x} h_{a} } \right)}} $$

where, $h_{a} = h_{1} + \alpha d_{1}$ is the corrected height of the hole, and $\alpha = 0.85$ is the correction factor^35^.

The impedance in the duct can be expressed as

3 $$ Z_{t} = \frac{{\rho_{0} c_{0} }}{{S_{e} }} $$

in which, $S_{e} = 1.072S_{d}$ is the equivalent area of the duct section, which is obtained from COMSOL simulations to account for the irregularity of the duct cross-section.

The acoustic pressure and volume velocity from the inlet to the first hole are

4 $$ p_{1} \left( x \right) = P_{1}^{ + } {\text{e}}^{{ - {\text{i}}k_{x} x}} + P_{1}^{ - } {\text{e}}^{{{\text{i}}k_{x} x}} ,U_{1} \left( x \right) = \frac{{P_{1}^{ + } {\text{e}}^{{ - {\text{i}}k_{x} x}} - P_{1}^{ - } {\text{e}}^{{{\text{i}}k_{x} x}} }}{{Z_{t} }} $$

where, $P_{1}^{ + }$ and $P_{1}^{ - }$ are the propagation coefficients in the directions of $+ x$ and $- x$.

At $x = 0$, we have

5 $$ p_{1} \left( 0 \right) = P_{1}^{ + } + P_{1}^{ - } ,U_{1} \left( 0 \right) = \frac{{P_{1}^{ + } - P_{1}^{ - } }}{{Z_{t} }} $$

So, Eq. (5) can be reduced to the following form

6 $$ \left[ {\begin{array}{*{20}c} {P_{1}^{ + } } \\ {P_{1}^{ - } } \\ \end{array} } \right] = {\mathbf{T}}_{1} \left[ {\begin{array}{*{20}c} {p_{1} \left( 0 \right)} \\ {U_{1} \left( 0 \right)} \\ \end{array} } \right] $$

where, ${\mathbf{T}}_{1}$ is the transfer matrix, is expressed as

7 $$ {\mathbf{T}}_{1} { = }\left[ {\begin{array}{*{20}c} \frac{1}{2} & {\frac{{Z_{t} }}{2}} \\ \frac{1}{2} & { - \frac{{Z_{t} }}{2}} \\ \end{array} } \right] $$

Acoustic pressure and volume velocity between the first and the second holes can be written as

8 $$ p_{2} \left( x \right) = P_{2}^{ + } {\text{e}}^{{ - {\text{i}}k_{x} \left( {x - D_{1} } \right)}} + P_{2}^{ - } {\text{e}}^{{{\text{i}}k_{x} \left( {x - D_{1} } \right)}} ,U_{2} \left( x \right) = \frac{{P_{2}^{ + } {\text{e}}^{{ - {\text{i}}k_{x} \left( {x - D_{1} } \right)}} - P_{2}^{ - } {\text{e}}^{{{\text{i}}k_{x} \left( {x - D_{1} } \right)}} }}{{Z_{t} }} $$

Appling the continuous conditions on acoustic pressure and volume velocity at $z = D_{1}$, we have

9 $$ p_{1} \left( {D_{1} } \right) = p_{2} \left( {D_{1} } \right),U_{1} \left( {D_{1} } \right) = U_{2} \left( {D_{1} } \right) + U_{d} \left( {D_{1} } \right) $$

where, $U_{d}$ is the velocity component of the resonator at $z = D_{1}$.

Substituting Eqs. (5) and (8) into Eq. (9), the following transfer matrix are obtained

10 $$ \left[ {\begin{array}{*{20}c} {P_{2}^{ + } } \\ {P_{2}^{ - } } \\ \end{array} } \right] = {\mathbf{T}}_{2} {\mathbf{N}}_{1} \left[ {\begin{array}{*{20}c} {P_{1}^{ + } } \\ {P_{1}^{ - } } \\ \end{array} } \right] $$

where

11 $$ {\mathbf{T}}_{2} { = }\left[ {\begin{array}{*{20}c} {\frac{{2 - Z_{t} /Z_{d} }}{2}} & {\frac{{ - Z_{t} /Z_{d} }}{2}} \\ {\frac{{Z_{t} /Z_{d} }}{2}} & {\frac{{2 + Z_{t} /Z_{d} }}{2}} \\ \end{array} } \right],{\mathbf{N}}_{1} { = }\left[ {\begin{array}{*{20}c} {{\text{e}}^{{ - {\text{i}}k_{x} D_{1} }} } & 0 \\ 0 & {{\text{e}}^{{{\text{i}}k_{x} D_{1} }} } \\ \end{array} } \right] $$

Similarly, we have the following transfer relationships

12 $$ \left[ {\begin{array}{*{20}c} {P_{3}^{ + } } \\ {P_{3}^{ - } } \\ \end{array} } \right] = {\mathbf{T}}_{2} {\mathbf{N}}_{2} \left[ {\begin{array}{*{20}c} {P_{2}^{ + } } \\ {P_{2}^{ - } } \\ \end{array} } \right],\left[ {\begin{array}{*{20}c} {P_{4}^{ + } } \\ {P_{4}^{ - } } \\ \end{array} } \right] = {\mathbf{T}}_{2} {\mathbf{N}}_{2} \left[ {\begin{array}{*{20}c} {P_{3}^{ + } } \\ {P_{3}^{ - } } \\ \end{array} } \right],\left[ {\begin{array}{*{20}c} {P_{5}^{ + } } \\ {P_{5}^{ - } } \\ \end{array} } \right] = {\mathbf{T}}_{2} {\mathbf{N}}_{2} \left[ {\begin{array}{*{20}c} {P_{4}^{ + } } \\ {P_{4}^{ - } } \\ \end{array} } \right] $$

where, $P_{3}^{ \pm }$ and $P_{4}^{ \pm }$ are the propagation coefficients between the resonator 2 and 3, 3 and 4, respectively. $P_{5}^{ \pm }$ is the propagation coefficient between the resonator 4 and the outlet of the duct, ${\mathbf{N}}_{2}$ is

13 $$ {\mathbf{N}}_{2} { = }\left[ {\begin{array}{*{20}c} {{\text{e}}^{{ - {\text{i}}k_{x} D_{2} }} } & 0 \\ 0 & {{\text{e}}^{{{\text{i}}k_{x} D_{2} }} } \\ \end{array} } \right] $$

At $x = A_{1}$, the relationship between $P_{5}^{ \pm }$ and transmitted acoustic pressure and velocity can be written as

14 $$ \left[ {\begin{array}{*{20}c} {p_{1} \left( {A_{1} } \right)} \\ {U_{1} \left( {A_{1} } \right)} \\ \end{array} } \right] = {\mathbf{T}}_{3} {\mathbf{N}}_{1} \left[ {\begin{array}{*{20}c} {P_{5}^{ + } } \\ {P_{5}^{ - } } \\ \end{array} } \right] $$

where

15 $$ {\mathbf{T}}_{3} { = }\left[ {\begin{array}{*{20}c} 1 & 1 \\ {\frac{1}{{Z_{t} }}} & { - \frac{1}{{Z_{t} }}} \\ \end{array} } \right] $$

Now, we have the following transfer relationship

16 $$ \left[ {\begin{array}{*{20}c} {p_{1} \left( {A_{1} } \right)} \\ {U_{1} \left( {A_{1} } \right)} \\ \end{array} } \right] = {\mathbf{T}}\left[ {\begin{array}{*{20}c} {p_{1} \left( 0 \right)} \\ {U_{1} \left( 0 \right)} \\ \end{array} } \right] $$

where, the transfer matrix ${\mathbf{T}} = {\mathbf{T}}_{3} {\mathbf{N}}_{1} \left( {{\mathbf{T}}_{2} {\mathbf{N}}_{2} } \right)^{3} {\mathbf{T}}_{2} {\mathbf{N}}_{1} {\mathbf{T}}_{1}$.

From Eq. (16), we have

17b $$ U_{1} \left( {A_{1} } \right) = \frac{{2\left( {t_{12} t_{21} - t_{11} t_{22} } \right)}}{{t_{21} Z_{d}^{2} - \left( {t_{11} + t_{22} } \right)Z_{d} + t_{12} }} $$

where, $t_{ij}$ is the corresponding element $(i,j)$ in ${\mathbf{T}}$.

Finally, the transmission coefficient $T$ of PMM is

18 $$ T = \frac{{2\left( {t_{12} t_{21} - t_{11} t_{22} } \right)Z_{t} }}{{t_{21} Z_{d}^{2} - \left( {t_{11} + t_{22} } \right)Z_{d} + t_{12} }} $$

**Figure 7:**
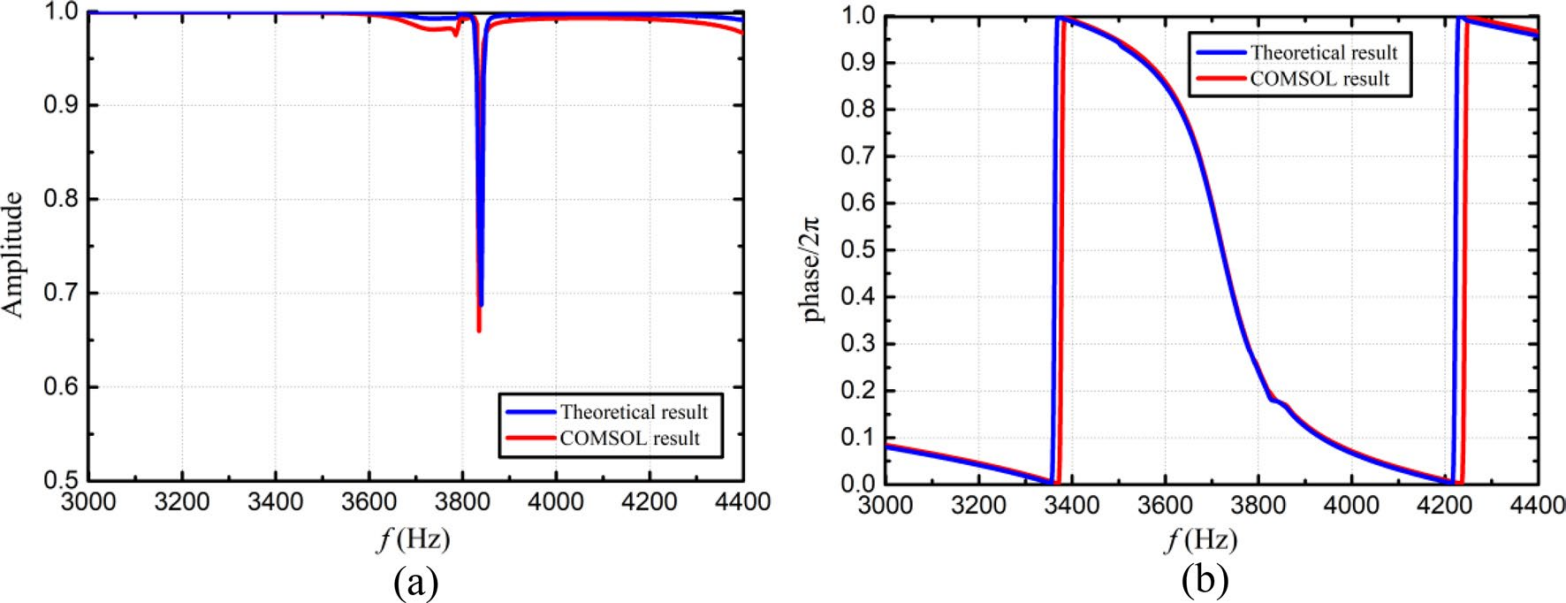
The transmission properties of the fundamental phase-modulating unit. (**a**) Amplitude versus frequency. (**b**) Phase shift versus frequency.

The transmission characteristics obtained by the above theoretical formulae are shown in Fig. 7. The simulation parameters are taken as follows: the hole diameter is $d_{1} = 3.6\,{\text{mm}}$, and the height is $h_{1} = 2\,{\text{mm}}$. The length, width and height of the backing cavity are $a_{1} = 10\,{\text{mm}}$, $b_{1} = 10\,{\text{mm}}$, and $l_{1} = 5\,{\text{mm}}$, respectively. The length, width and height of fundamental phase-modulating metasurface are $A_{1} = 45{\text{mm}}$, $B_{1} = 12\,{\text{mm}}$ and $L_{1} = 8\,{\text{mm}}$, respectively. The distance from the hole center to the boundary is $D_{1} = 6\,{\text{mm}}$, the distance between two adjacent holes is $D_{2} = 10\,{\text{mm}}$. The radius of the duct is $R = 50\,{\text{mm}}$ and the central angle is $\theta = 45^{ \circ }$. As depicted in Fig. 7, the theoretical results obtained from Eq. (18) exhibit good agreements with the COMSOL predictions, thus confirming the effectiveness of the theoretical method.

**Figure 8:**
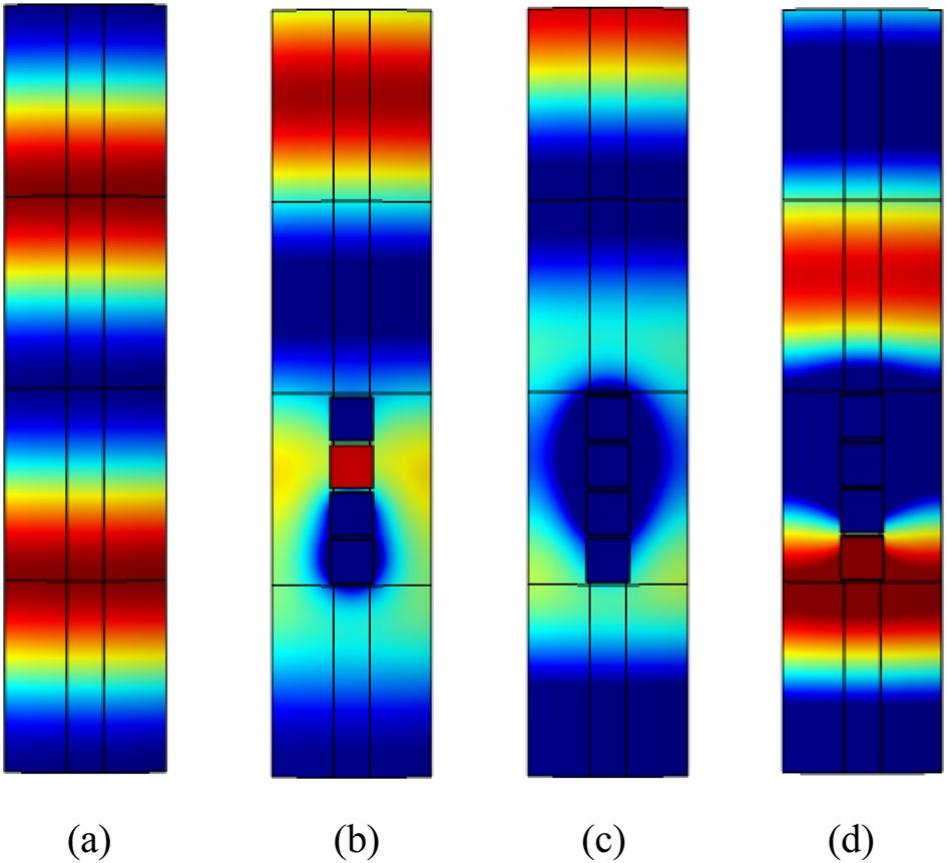
Acoustic pressure field of the fundamental phase-modulating unit. (**a**) Unit 1. (**b**) Unit 2. (**c**) Unit 3. (**d**) Unit 4.

**Figure 9:**
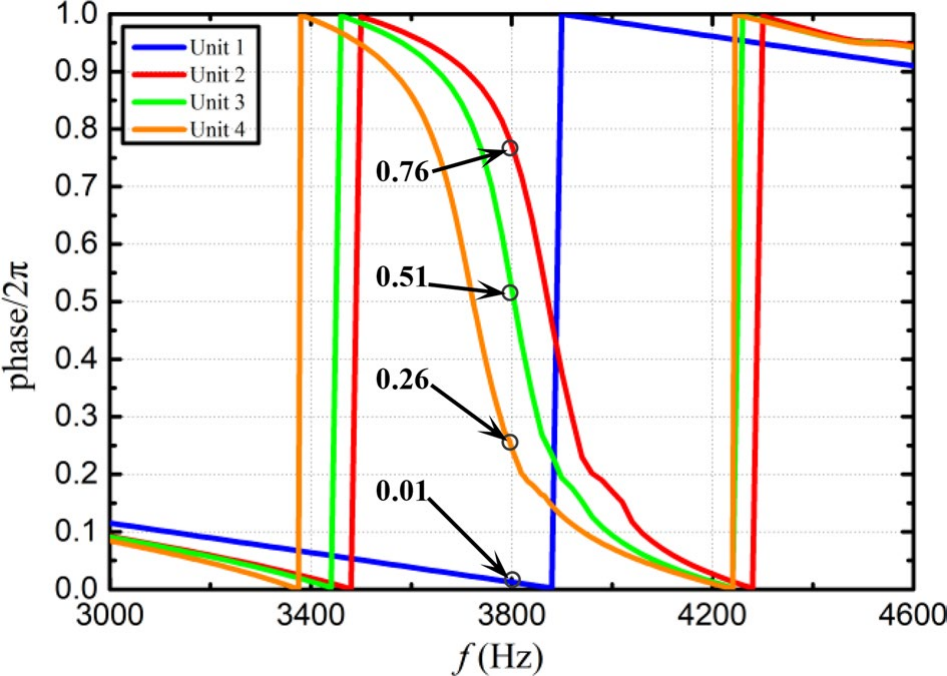
Transmitted phase shifts produced by unit 1–4.

**Figure 10:**
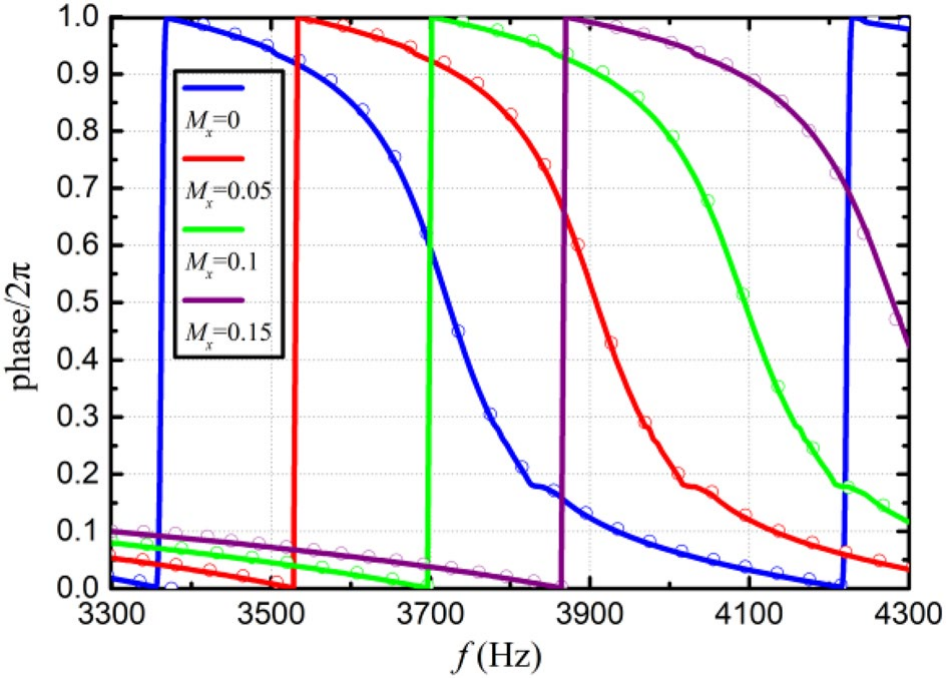
Transmitted phase shifts produced by unit 2 for different grazing flow Mach numbers, the theoretical results are drawn with solid lines and the COMSOL results are drawn with circular lines.

**Figure 11:**
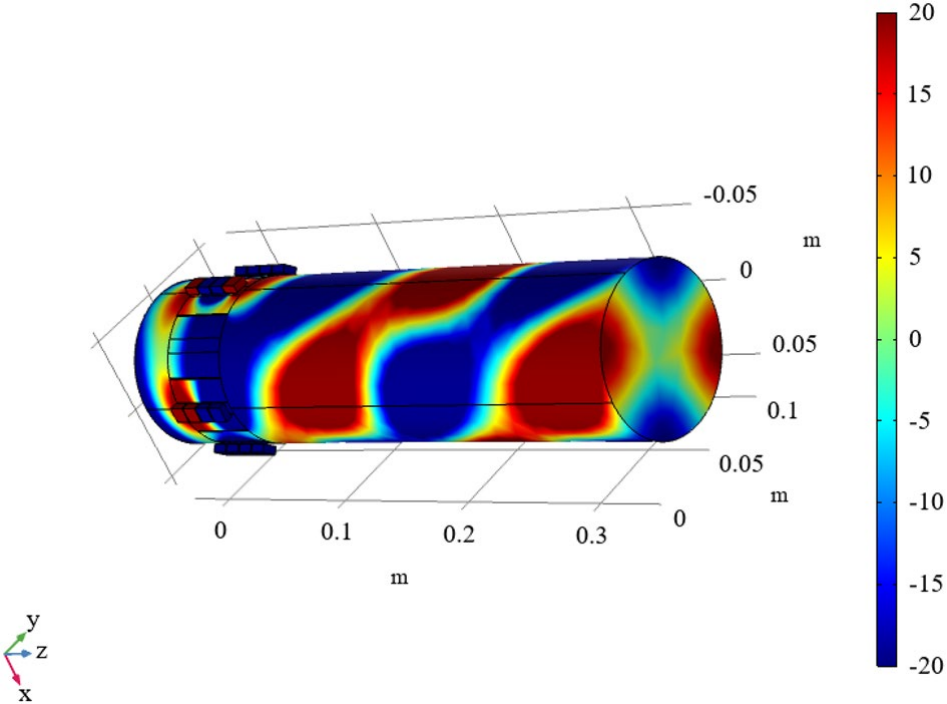
Acoustic pressure field (Pa) of the generated spinning wave.

In order to ensure that each unit has a specified phase shift, optimal design of the metasurface was performed by genetic algorithm using with the theoretical formulas given in this section. Note that unit 1 employs a null structure, hence only unit 2–4 need to be optimized. The optimization variable is taken as the hole diameter of the unit. Assuming that the four Helmholtz resonators in one unit have the same hole diameter, hence a total of three optimization variables are used. In addition, during the optimization process, except for design variables, all the other parameters remain unchanged and their values are the same as those used in Fig. 7. The objective of optimization is to minimize the discrepancy between transmission phase shift and a predetermined value. Optimization was carried out at an incident wave frequency of 3800Hz, the population size and the mutation rate are taken as 20 and 0.2, respectively. The optimization process terminates when after 1500 generations. At this time, the transmittance is kept above 95%. The optimized hole diameters of unit 2–4 are $d_{12} = 3.748\,{\text{mm}}$, $d_{13} = 3.841\,{\text{mm}}$ and $d_{14} = 3.949\,{\text{mm}}$, respectively. The acoustic pressures and phase shifts generated by the optimized PMM are illustrated in Figs. 8 and 9, respectively. It can be seen that for the incident wave frequency of 3800Hz, the phase shifts (multiples of ${2}\pi$) generated by unit 1–4 are 0.01, 0.76, 0.51 and 0.26, respectively. When converted to multiples of $\pi {/2}$, the phase shifts of unit 1–4 are 0.04, 3.04, 2.04 and 1.04, respectively. As expected, the phase difference of $\pi {/2}$ at 3800Hz between two adjacent units is successfully achieved. In addition, the phase shifts under different grazing flow Mach numbers are shown in Fig. 10, in which we can see that the phase curves shift to the higher frequency with the increase of Mach number. To verify the effectiveness of the spinning wave generated by the optimized PMM structure, the COMSOL simulations were conducted with an incident plane wave at 3800 Hz. The pressure acoustic physical field is adopted, the sound pressure of the incident wave is 20 Pa and the direction of propagation is the positive z-axis. The calculated acoustic pressure field is presented in Fig. 11. As expected, the plane wave is successfully transformed into a spinning form at the exit of PMM.

## Acoustic liner in the duct

### Theoretical predictions of noise-reduction characteristics of ALD

**Figure 12:**
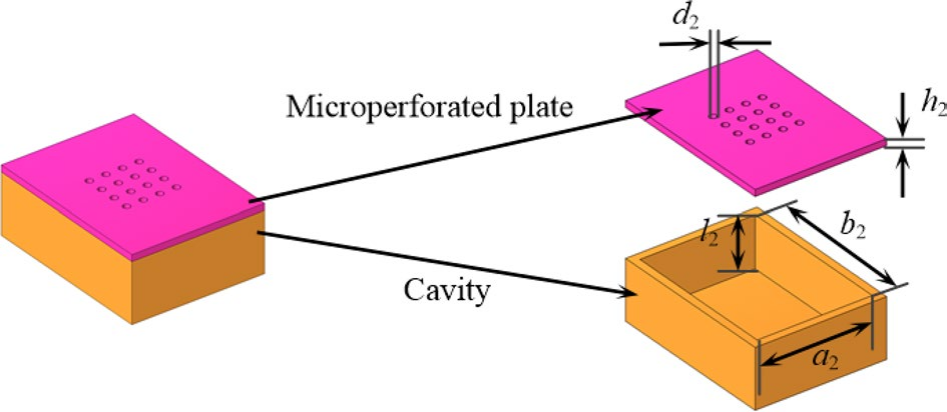
The fundamental resonance element of the acoustic absorber.

**Figure 13:**
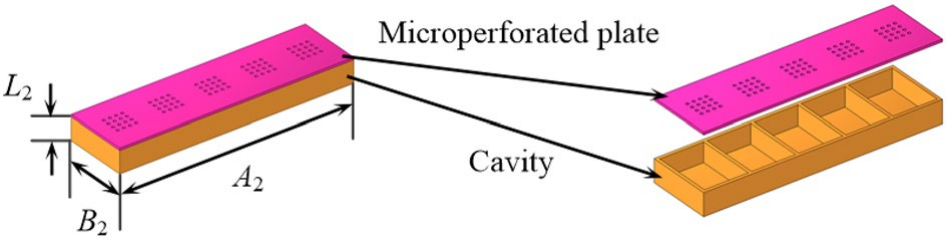
The acoustic absorption unit of ALD.

The fundamental resonance element of ALs consists of a micro-perforated plate with 16 holes and an enclosed backing cavity, as illustrated in Fig. 12. The diameter and height of the hole in perforated plate are $d_{2}$, and $h_{2}$, respectively. The length, width and height of the backing cavity are $a_{2}$, $b_{2}$, and $l_{2}$, respectively. A total of five fundamental resonance elements, named acoustic absorption unit, are arranged longitudinally along the duct, as illustrated in Fig. 13.

The acoustic field within the duct is governed by the wave equation^36^

19 $$ \left( {{\text{i}}k + M_{x} \frac{\partial }{\partial x}} \right)^{2} p\left( {r,\theta ,x} \right) = \nabla^{2} p\left( {r,\theta ,x} \right) $$

where, $p$ is the acoustic pressure. In a polar coordinate system, $k$ is the acoustic wave number, the radial distance and angle of the duct section are denoted by $r$ and $\theta$ respectively, while $x$ represents axial coordinate, $M_{x}$ is the Mach number of the grazing flow.

Separating the variables $r$, $\theta$ and $x$, modal solutions of Eq. (19) can be expressed as^37^

20 $$ \begin{aligned} p\left( {r,\theta ,x} \right) & = \sum\limits_{m = - \infty }^{\infty } {\sum\limits_{n = 1}^{\infty } {\left( {p_{m,n}^{ + } + p_{m,n}^{ - } } \right)} } \\ & = \sum\limits_{m = - \infty }^{\infty } {\sum\limits_{n = 1}^{\infty } {\left[ {A_{m,n}^{ + } J_{m} \left( {\kappa_{1m,n}^{ + } r} \right)e^{{i\left( {m\theta - k_{1m,n}^{ + } x} \right)}} + A_{m,n}^{ - } J_{m} \left( {\kappa_{1m,n}^{ - } r} \right)e^{{i\left( {m\theta - k_{1m,n}^{ - } x} \right)}} } \right]} } \\ \end{aligned} $$

where, $m$ and $n$ are the circumferential and radial modes, respectively.$k_{1}$ and $\kappa_{1}$ are the axial and circumferential wave numbers of the rigid wall sections, respectively. Symbols “$+$”, “$-$” represent the running modes to the right and left, respectively. $J_{m}$ is the *m*th order Bessel function, $A_{m,n}$ is the amplitude of the $(m,n)$ mode. $k_{1m,n}^{ \pm }$ can be written as

21 $$ k_{1m,n}^{ \pm } = \frac{k}{{1 - M_{x}^{2} }}\left( { - M_{x} \pm \sqrt {1 - \left( {1 - M_{x}^{2} } \right)\left( {\frac{{\kappa_{1m,n}^{ \pm } }}{k}} \right)^{2} } } \right) $$

A modal solution of Eq. (20) is

22 $$ p_{m,n} \left( {r,\theta ,x} \right) = A_{m,n}^{ + } J_{m} \left( {\kappa_{1m,n} r} \right){\text{e}}^{{{\text{i}}\left( {m\theta - k_{1m,n} x} \right)}} $$

The boundary condition on the wall of the duct $(r = R)$ in the ALD section is

23 $$ \frac{\partial p}{{\partial r}} = - \frac{{{\text{i}}k}}{{Z_{l} }}\left( {1 - \frac{{{\text{i}}M_{x} }}{k}\frac{\partial }{\partial x}} \right)^{2} p $$

where, $R$ is the radius of the duct.

In which, the specific acoustic impedance of the ALD $Z_{l}$ is expressed as^38^

24 $$ Z_{l} = \frac{{\rho_{0} c_{0} }}{\sigma }\left[ {\frac{{\sqrt {8\mu \omega } }}{{c_{0} }}\left( {1 + \frac{{h_{2} }}{{d_{2} }}} \right) + \frac{8\mu }{{d_{2} c_{0} }} + \frac{{\pi^{2} }}{2}\left( {\frac{{d_{2} }}{\lambda }} \right)^{2} } \right] + \frac{{{\text{i}}\rho_{0} \omega }}{\sigma }\left[ {1 + \frac{{8d_{2} (1 - 0.7\sqrt \sigma )}}{3\pi }} \right] - {\text{i}}\frac{{\rho_{0} c_{0} }}{{kV_{2} }} $$

where, $\lambda$ is the acoustic wavelength, $\sigma$ the perforation rate, $\mu$ the dynamic viscosity of air, $V_{2} = a_{2} b_{2} l_{2}$ the volume of the cavity.

From Eqs. (22) and (23), the following eigenvalue problem can be obtained

25 $$ \kappa_{m,n} R\frac{{J^{\prime}_{m} (\kappa_{1m,n} R)}}{{J_{m} (\kappa_{1m,n} R)}} = - \frac{{{\text{i}}kR}}{{Z_{l} }}\left( {1 - M_{x} \frac{{k_{1m,n} }}{k}} \right)^{2} $$

For the rigid wall section, the circumferential wave number can be obtained by solving the following nonlinear equation

26 $$ J^{\prime}_{m} \left( {\kappa_{1m,n} R} \right) = 0 $$

In a rigid duct, only a limited number of modes can propagate and transfer acoustic power at a fixed frequency, while the remaining modes are truncated. The cut-off ratio of different modes are defined as $g_{m,n} = k/\kappa_{1m,n} \sqrt {1 - M_{x}^{2} }$. If $g_{m,n} > 1$, the mode $\left( {m,n} \right)$ is cut-on and can propagate in duct. The frequency corresponding to $g_{m,n} = 1$ is called cut-off frequency and is shown in Table 1 for each mode $\left( {m,n} \right)$. It can be seen that only modes $\left( {0,1} \right)$, $\left( {1,1} \right)$ and $\left( {2,1} \right)$ can propagate under the condition of the duct radius of 0.05m and the incident wave of 3800Hz in this paper, and all modes are truncated except for the first order radial mode. Therefore $n = 1$ is utilized in subsequent derivation.

**Table 1:** Cut-off frequencies of different modes.

$\left( {m,n} \right)$	Cut-off frequency (Hz)
(0,1)	0
(1,1)	2032
(2,1)	3345
(3,1)	4587
(0,2)	4183
(1,2)	5834
(2,2)	7315
(3,2)	8751

Acoustic scattering may arise from variations in the wall impedance of a duct. The rigid and impedance boundaries are represented as a superposition of Fourier–Bessel modes^33^, encompassing right $( + )$ and left $( - )$ modes. For the circumferential mode $m$, we have

27a $$ p_{m}^{1} \left( {r,\theta ,x} \right) = \left[ {A_{m,1}^{1 + } J_{m} \left( {\kappa_{1m,1}^{ + } r} \right){\text{e}}^{{ - {\text{i}}k_{1m,1}^{ + } x}} + A_{m,1}^{1 - } J_{m} \left( {\kappa_{1m,1}^{ - } r} \right){\text{e}}^{{ - {\text{i}}k_{1m,1}^{ - } x}} } \right]{\text{e}}^{{{\text{i}}m\theta }} $$

27b $$ p_{m}^{2} \left( {r,\theta ,x} \right) = \left[ {A_{m,1}^{2 + } J_{m} \left( {\kappa_{2m,1}^{ + } r} \right){\text{e}}^{{ - {\text{i}}k_{1m,1}^{ + } x}} + A_{m,1}^{2 - } J_{m} \left( {\kappa_{2m,1}^{ - } r} \right){\text{e}}^{{ - {\text{i}}k_{2m,1}^{ - } x}} } \right]{\text{e}}^{{{\text{i}}m\theta }} $$

27c $$ p_{m}^{3} \left( {r,\theta ,x} \right) = \left[ {A_{m,1}^{3 + } J_{m} \left( {\kappa_{1m,1}^{ + } r} \right){\text{e}}^{{ - {\text{i}}k_{1m,1}^{ + } x}} + A_{m,1}^{3 - } J_{m} \left( {\kappa_{1m,1}^{ - } r} \right){\text{e}}^{{ - {\text{i}}k_{1m,1}^{ - } x}} } \right]{\text{e}}^{{{\text{i}}m\theta }} $$

where, $\kappa_{2}$ and $k_{2}$ are the axial and circumferential wave numbers of the ALD section, respectively.

The inlet and outlet sections are characterized as rigid wall boundaries, while the acoustic absorption section is applied the impedance boundary. The axial component of acoustic velocity in each section can be determined through application of the momentum equation

28 $$ u_{1m,1}^{ \pm } = \frac{{q_{1m,1}^{ \pm } p_{1m,1}^{ \pm } }}{{\rho_{0} c_{0} }},u_{2m,1}^{ \pm } = \frac{{q_{2m,1}^{ \pm } p_{2m,1}^{ \pm } }}{{\rho_{0} c_{0} }} $$

where, $$q_{1m,1}^{ \pm } = k_{1m,1}^{ \pm } /\left( {k - k_{1m,1}^{ \pm } M_{x} } \right),q_{2m,1}^{ \pm } = k_{2m,1}^{ \pm } /\left( {k - k_{2m,1}^{ \pm } M_{x} } \right)$$.

Note that $A_{m,1}^{1 + }$ is the amplitude of the inlet incident acoustic source and is a known quantity. The reflection amplitude at the exit defaults to 0, i.e., $A_{m,1}^{3 - } = 0$. To determine the amplitude of other sections, it is necessary to ensure acoustic pressure and velocity continuity at the junction of each region by matching them accordingly.

29a $$ \int_{r = 0}^{R} {rJ_{m} \left( {\kappa_{1m,1} r} \right)\left[ {p_{m}^{2 + } \left( {r,\theta ,D_{3} } \right) - p_{m}^{1 - } \left( {r,\theta ,D_{3} } \right)} \right]{\text{d}}r = 0} $$

29b $$ \int_{r = 0}^{R} {rJ_{m} \left( {\kappa_{1m,1} r} \right)\left[ {u_{m}^{2 + } \left( {r,\theta ,D_{3} } \right) - u_{m}^{1 - } \left( {r,\theta ,D_{3} } \right)} \right]{\text{d}}r = 0} $$

29c $$ \int_{r = 0}^{R} {rJ_{m} \left( {\kappa_{1m,1} r} \right)\left[ {p_{m}^{3 + } \left( {r,\theta ,D_{3} + A_{2} } \right) - p_{m}^{2 - } \left( {r,\theta ,D_{3} + A_{2} } \right)} \right]{\text{d}}r = 0} $$

29d $$ \int_{r = 0}^{R} {rJ_{m} \left( {\kappa_{1m,1} r} \right)\left[ {u_{m}^{3 + } \left( {r,\theta ,D_{3} + A_{2} } \right) - u_{m}^{2 - } \left( {r,\theta ,D_{3} + A_{2} } \right)} \right]{\text{d}}r = 0} $$

where, $D_{3}$ is the distance between the inlet of the duct and the ALD section.

Converting Eq. (29) into matrix form, we obtain

30a $$ \left[ {\frac{{A_{m,1}^{2 + } }}{{A_{m,1}^{1 - } }}} \right] = {\mathbf{E}}_{1} {\mathbf{D}}_{1} \left[ {\frac{{A_{m,1}^{1 + } }}{{A_{m,1}^{2 - } }}} \right] $$

30b $$ \left[ {\frac{{A_{m,1}^{3 + } }}{{A_{m,1}^{2 - } }}} \right] = {\mathbf{E}}_{2} {\mathbf{D}}_{2} \left[ {\frac{{A_{m,1}^{2 + } }}{{A_{m,1}^{3 - } }}} \right] $$

where, ${\mathbf{E}}_{1}$, ${\mathbf{E}}_{2}$ are denoted by

31a $$ {\mathbf{E}}_{1} = \left[ {\begin{array}{*{20}c} {S_{2}^{ + } } & { - S_{1} } \\ {V_{2}^{ + } } & { - V_{1}^{ - } } \\ \end{array} } \right]^{ - 1} \left[ {\begin{array}{*{20}c} {S_{1} } & { - S_{2}^{ - } } \\ {V_{1}^{ + } } & { - V_{2}^{ - } } \\ \end{array} } \right] $$

31b $$ {\mathbf{E}}_{2} = \left[ {\begin{array}{*{20}c} {S_{1}^{ + } } & { - S_{2}^{ - } } \\ {S_{1}^{ + } } & { - S_{2}^{ - } } \\ \end{array} } \right]^{ - 1} \left[ {\begin{array}{*{20}c} {S_{2}^{ + } } & { - S_{1}^{ - } } \\ {S_{2}^{ + } } & { - S_{1}^{ - } } \\ \end{array} } \right] $$

with

32a $$ S_{1} = \int_{r = 0}^{R} {rJ_{m} \left( {\kappa_{1m,1} r} \right)^{2} } {\text{d}}r,S_{2}^{ \pm } = \int_{r = 0}^{R} {rJ_{m} \left( {\kappa_{1m,1} r} \right)J_{m} \left( {\kappa_{2m,1}^{ \pm } r} \right)} {\text{d}}r $$

32b $$ V_{1}^{ \pm } = \frac{{q_{1m,1}^{ \pm } S_{1} }}{{\rho_{0} c_{0} }},V_{2}^{ \pm } = \frac{{q_{2m,1}^{ \pm } S_{2}^{ \pm } }}{{\rho_{0} c_{0} }} $$

Equation (32a) can be solved analytically, given by

33a $$ \int_{r = 0}^{R} {rJ_{m} \left( {\kappa_{1m,1} r} \right)J_{m} \left( {\kappa_{1m,1} r} \right)} {\text{d}}r = \left\{ {\begin{array}{*{20}l} {\frac{{R^{2} }}{2}\left[ {1 - \left( {\frac{m}{{\kappa_{1m,1} R}}} \right)^{2} } \right],} \hfill & {\quad i = j} \hfill \\ {0,} \hfill & {\quad i \ne j} \hfill \\ \end{array} } \right. $$

33b $$ \int_{r = 0}^{R} {rJ_{m} \left( {\kappa_{1m,1} r} \right)J_{m} \left( {\kappa_{2m,1}^{ \pm } r} \right)} {\text{d}}r = \frac{R}{{\kappa_{1m,1}^{2} - \kappa_{2m,1}^{ \pm 2} }}\left[ {\kappa_{1m,1} J_{m + 1} \left( {\kappa_{1m,1} R} \right)J_{m} \left( {\kappa_{2m,1}^{ \pm } R} \right) - \kappa_{2m,1}^{ \pm } J_{m} \left( {\kappa_{1m,1} R} \right)J_{m + 1} \left( {\kappa_{2m,1}^{ \pm } R} \right)} \right] $$

Matrices ${\mathbf{D}}_{1}$, ${\mathbf{D}}_{2}$ are expressed as

34a $$ {\mathbf{D}}_{1} = \left[ {\begin{array}{*{20}c} {{\text{e}}^{{ - {\text{i}}k_{1m,1}^{ + } D_{3} }} } & {} \\ {} & {{\text{e}}^{{{\text{i}}k_{2m,1}^{ - } A_{2} }} } \\ \end{array} } \right] $$

34b $$ {\mathbf{D}}_{2} = \left[ {\begin{array}{*{20}c} {{\text{e}}^{{ - {\text{i}}k_{2m,1}^{ + } A_{2} }} } & {} \\ {} & {{\text{e}}^{{{\text{i}}k_{1m,1}^{ - } D_{3} }} } \\ \end{array} } \right] $$

From Eq. (30), the relationship between $A_{m,1}^{1 + }$ and $A_{m,1}^{3 + }$ can be finally obtained. In the section with rigid walls, the total acoustic power is obtained by summing up the acoustic power in all transmission modes. The modal acoustic power $W_{m,1}^{ \pm }$ can be mathematically expressed as follows

35 $$ W_{m,1}^{ \pm } = \left| {A_{m,1}^{ \pm } } \right|^{2} \frac{{\pi b^{2} }}{{2\rho_{0} c_{0} }}\left| {J_{m} \left( {\kappa_{1m,1} R} \right)} \right|^{2} \left[ {1 - \left( {\frac{m}{{\kappa_{1m,1} R}}} \right)^{2} } \right]\left[ {\left( {1 + M_{x}^{2} } \right){\text{Re}} \left\{ {q_{m,1}^{ \pm } } \right\} + M_{x} \left( {1 + |q_{m,1}^{ \pm } |^{2} } \right)} \right] $$

Assuming that all acoustic energy is contained in the first-order radial mode at the entrance, the transmission loss of acoustic energy can be defined as

36 $$ {\text{TL}} = 10\lg \frac{{W_{m,1}^{1 + } }}{{\sum\limits_{m} {W_{m,1}^{3 + } } }} $$

Further analysis indicates that mode $\left( {m,n} \right) = \left( {2,1} \right)$ exhibits a transmitted acoustic power level of $1.58 \times 10^{ - 3} \,{\text{W}}$ at 3800 Hz, which is much higher than the other modes, as shown in Table 2. It is further demonstrated that the PMM section can transform plane waves into incident waves of (2,1) mode.

**Table 2:** Acoustic power level of different modes.

$\left( {m,n} \right)$	Acoustic power level (W)
(0,1)	3.65 × 10^−10^
(1,1)	4.68 × 10^−9^
(2,1)	1.58 × 10^−3^

### Optimization design of the acoustic absorbing structure

The radius of ALD structure is set to $R = 50\,{\text{mm}}$, and the length, width of the acoustic absorption unit in Fig. 13 are set to $A_{2} = 76\,{\text{mm}}$, $B_{2} = 21\,{\text{mm}}$, respectively. The length and width of the cavity are $a_{2} = 14\,{\text{mm}}$ and $b_{2} = 19\,{\text{mm}}$, respectively. The number of cavities along the axial and circumferential directions is 5 and 16, respectively. Genetic algorithm is used to obtain the optimized structural parameters of acoustic absorption segment of the PMM-ALD structure. The objective of optimization is to maximize the transmission loss at 3800 Hz. The optimization variables are the aperture of the microperforated plate and the height of the cavity. The population size and the mutation rate are taken as 20 and 0.2, respectively. The optimization process terminates when after 1500 generations. The constraint condition is that the radial dimension of the acoustic absorbing structure does not exceed the PMM thickness. The optimized structural parameters are as follows: the hole diameter and the height of the perforated plate are $d_{2} = 0.88\,{\text{mm}}$ and $h_{2} = 0.796\,{\text{mm}}$, respectively, the height of the cavity is $l_{2} = 6.11\,{\text{mm}}$. The traditional ALD structure is optimized using the same algorithm as above, and the optimized structural parameters are as follows: the hole diameter and the height of the perforated plate are $d_{2} = 0.85\,{\text{mm}}$ and $h_{2} = 0.823\,{\text{mm}}$, respectively, the height of the cavity is $l_{2} = 6.62\,{\text{mm}}$. In addition, the total length of the traditional ALD structure is the same as the sum of the lengths of the PMM and ALD segments for the PMM-ALD structure. After optimization, the PMM-ALD structure and the traditional ALD structure have the same resonance frequency of 3800 Hz.

**Figure 14:**
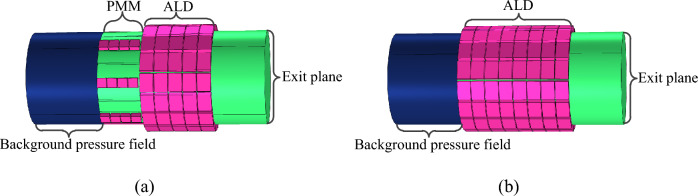
The noise-reduction structure. (**a**) Proposed PMM-ALD structure. (**b**) Traditional ALD structure.

To demonstrate the advantages of the proposed noise-reduction structure, we also designed a traditional ALD structure consisting of only acoustic absorbers as a contrast, as shown in Fig. 14. For the purpose of comparison, the axial dimension of the traditional ALD structure is designed as the sum of the PMM and ALD lengths of the PMM-ALD structure. The corresponding simulation results are given in Section “Simulation results”.

## Simulation results

**Figure 15:**
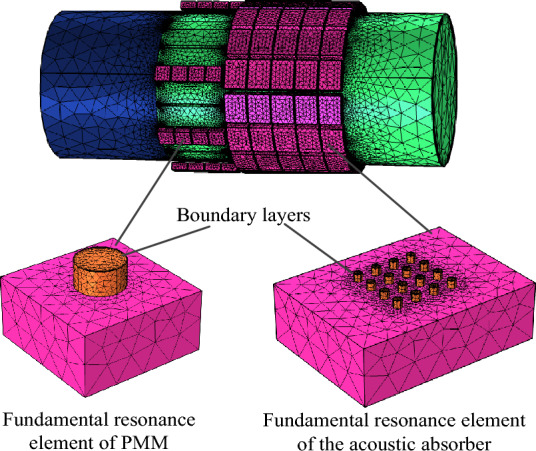
Finite element meshes of the PMM-ALD structure.

In this section, the noise-reduction performances of the proposed structure are validated through COMSOL Multiphysics platform. In acoustic simulations, the inlet section is modeled as the background pressure field. The incident plane wave has a frequency of 3300–4300Hz and an amplitude of 20 Pa. According to Table 1, the port boundary conditions with modes from (0,1) to (3,2) are implemented at both the inlet and outlet sections of the duct, which encompass all modes where reflected and transmitted waves occur. The remaining boundaries are subject to hard boundary conditions. Figure 15 illustrates the schematic of acoustic finite element meshes, wherein boundary layers are adopted on the duct wall and hole wall to account for viscosity effects. In simulations, the number of boundary layers is taken as 6 and the stretch factor of boundary layers is 1.2. The free tetrahedral meshes are used for the rest of the structure, the maximum and minimum element sizes are $0.01\,{\text{m}}$ and $5.5 \times 10^{ - 4} \,{\text{m}}$.

**Figure 16:**
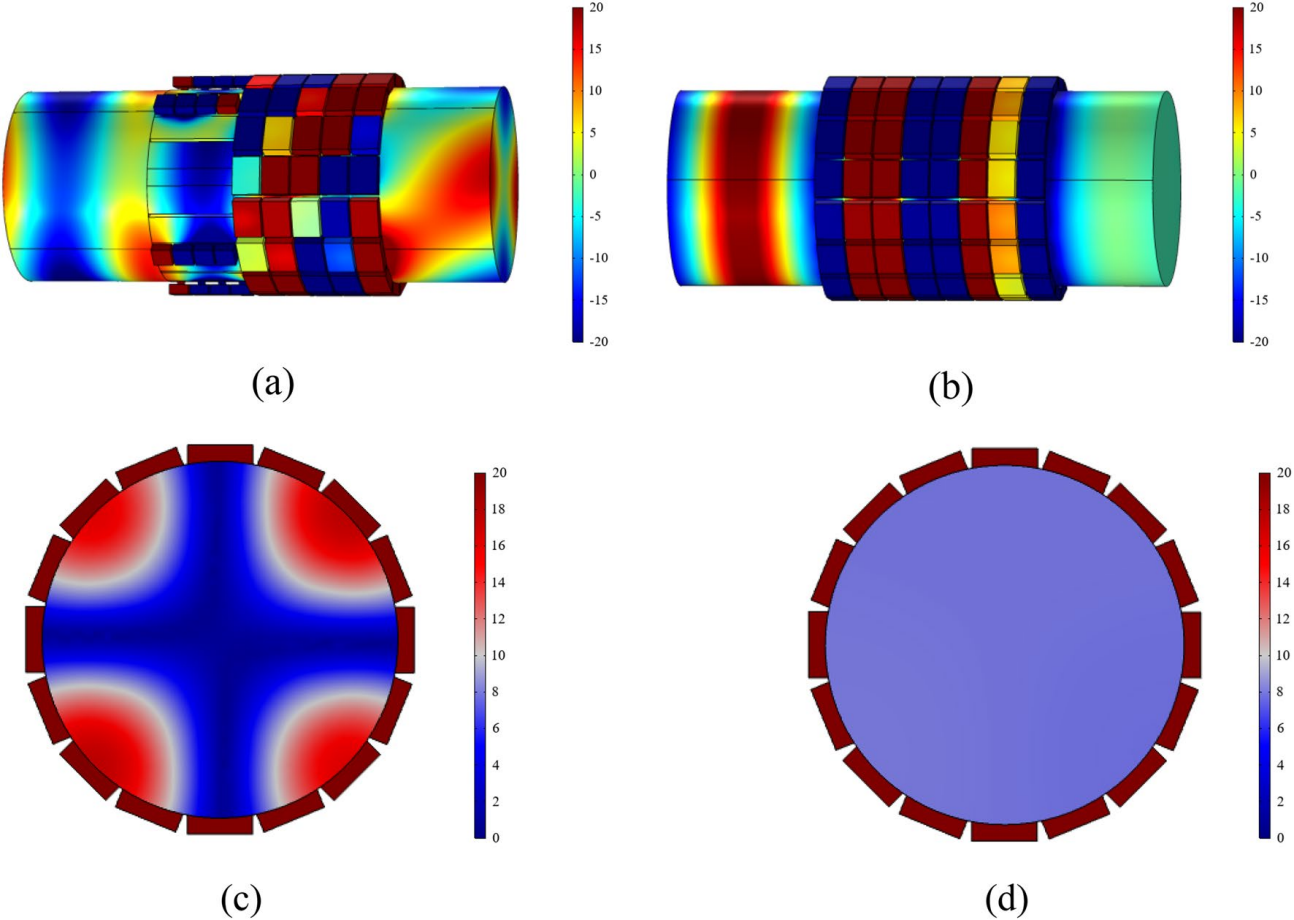
Acoustic pressure fields of the noise-reduction structure (Pa). (**a**) Acoustic pressure field of the PMM-ALD structure. (**b**) Acoustic pressure field of the traditional ALD structure. (**c**) Absolute value of acoustic pressure field at the exit plane of the PMM-ALD structure. (**d**) Absolute value of acoustic pressure field at the exit plane of the traditional ALD structure.

**Figure 17:**
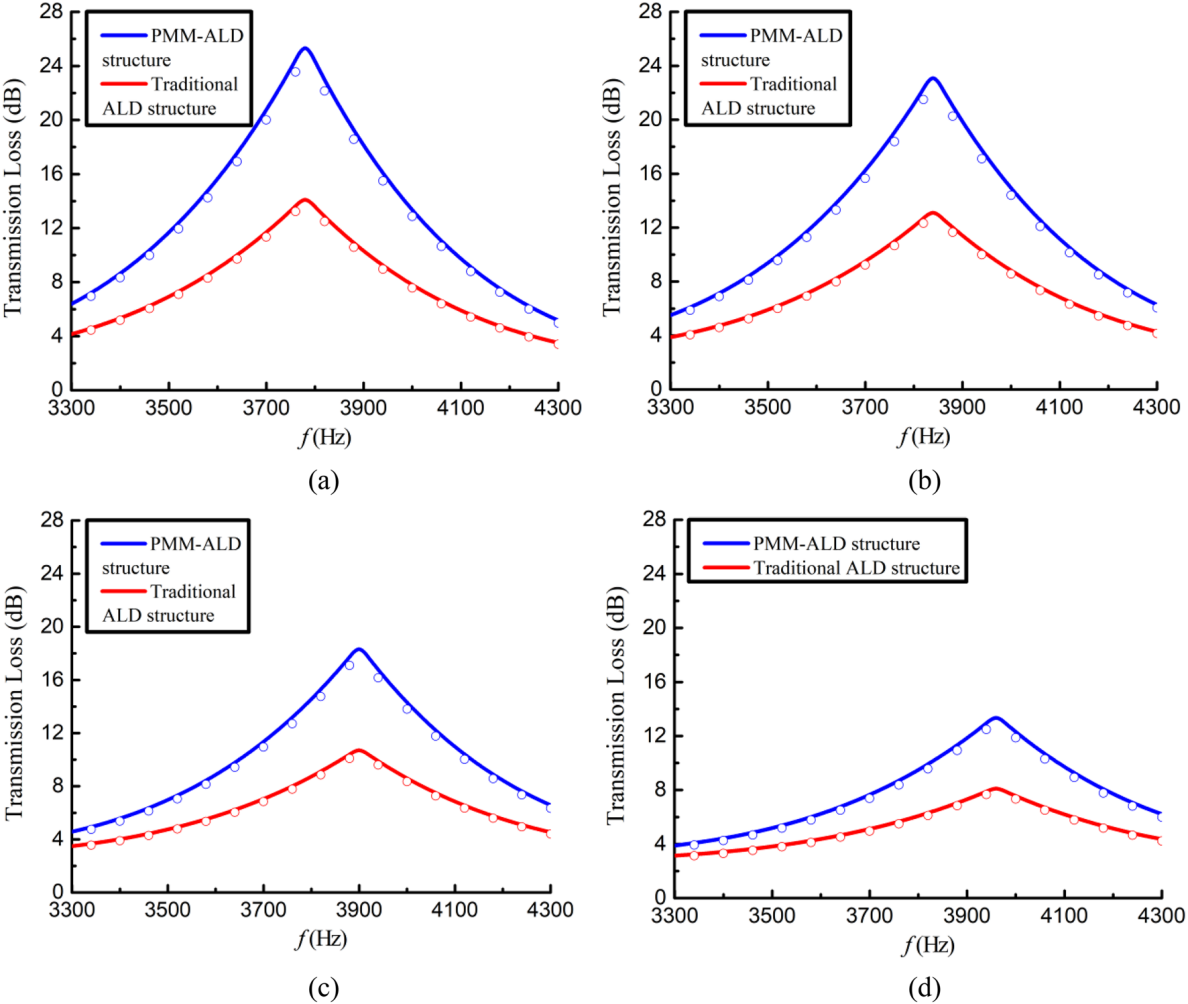
Transmission loss of the noise-reduction structure for different grazing flow Mach numbers. The theoretical results are drawn with solid lines and the COMSOL results are drawn with circular lines. (**a**) $M_{x} = 0$. (**b**) $M_{x} = 0.05$. (**c**) $M_{x} = 0.1$. (**d**) $M_{x} = 0.15$.

The predicted acoustic pressure fields of both structures are depicted in Fig. 16. Different form the traditional ALD (see Fig. 16b), it can be observed form Fig. 16a that the spinning wave is formed after passing through the PMM section and subsequently absorbed by the ALD section. By comparing the outlet sections depicted in Fig. 16c and d, it can be inferred that the PMM-ALD structure exhibits a higher degree of pressure concentration along the duct wall, whereas the traditional ALD structure tends to a uniform pressure distribution. Figure 17 gives transmission losses of the PMM-ALD and the traditional ALD structures obtained by theoretical calculations and COMSOL simulations. It can be seen that the results obtained by two methods are in good agreement, and the transmission loss of the proposed structure is much higher than that of the traditional one near the designed frequency of 3800 Hz. As indicated by Eqs. (21) and (25), an increase in circumferential mode *m* results in an increase in circumferential wave number $\kappa_{m,n}$ and a decrease in axial wave number $k_{m,n}$. As a result, the axial velocity component of acoustic waves in the duct decreases, leading to a prolonged contact time of spinning wave with the ALD structure. In this way, the overall noise-reduction performance is improved.

**Figure 18:**
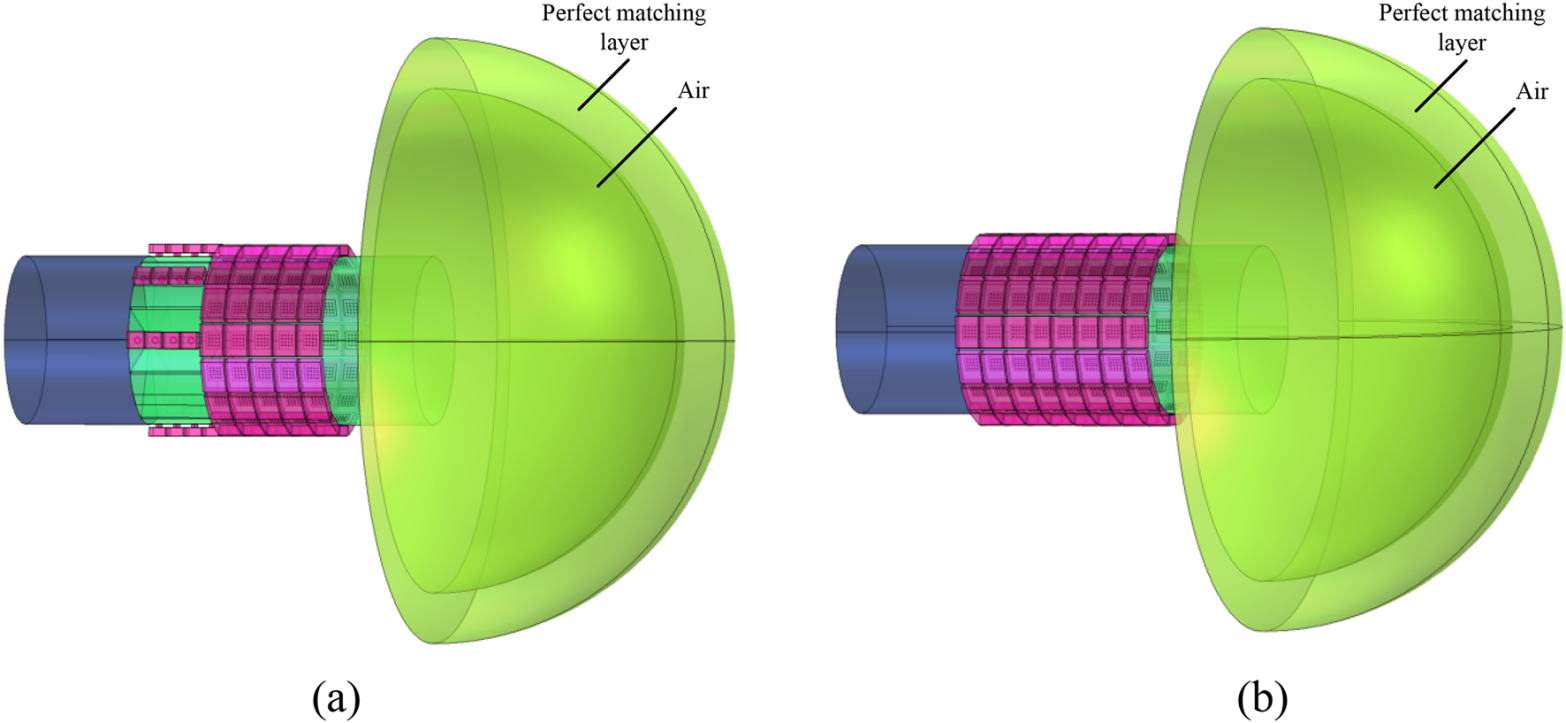
Far-field calculation diagrams of the noise-reduction structures. (**a**) PMM-ALD structure. (**b**) Traditional ALD structure.

**Figure 19:**
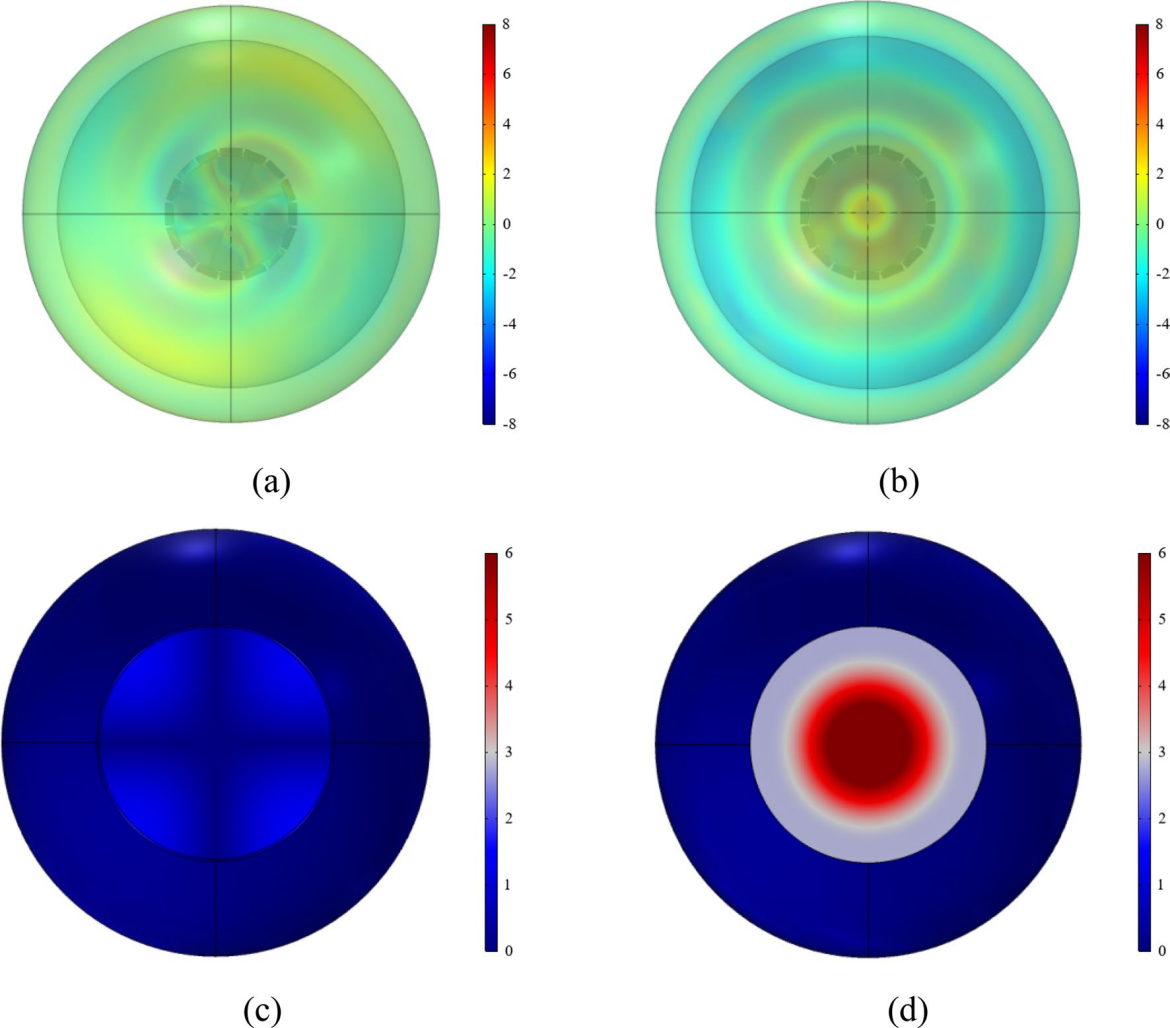
The far-field acoustic pressure (Pa). (**a**) PMM-ALD structure. (**b**) Traditional ALD structure. (**c**) Absolute value of internal acoustic pressure of the PMM-ALD structure. (**d**) Absolute value of internal acoustic pressure of the traditional ALD structure.

In addition, after the acoustic wave leaves the duct, the spinning wave exhibits a more uniform scattering compared to the plane wave, which results in a further reduction of the acoustic energy per unit area. To demonstrate this, the far-field acoustic pressure is calculated and is shown in Fig. 18. In simulations, the radius of the far-field hemisphere is taken as 0.3 m. The boundary condition of perfect matching layer is applied in the far-field. The predicted acoustic pressure field on the hemisphere in the absence of grazing flow is illustrated in Fig. 19. According to Fig. 19a,b, it is evident that acoustic waves exhibit a helical divergent propagation pattern upon exiting the PMM-ALD structure, whereas they propagate in a concentrated horizontal manner for the traditional ALD structure. Figure 19c,d displays the absolute value of internal sound pressure within a hole with diameter of 0.2m excavated at the far field boundary. By comparing the results of Fig. 19c,d, we can observe that the far-field acoustic pressure per unit area of the PMM-ALD structure is significantly lower than that of the traditional ALD structure.

**Figure 20:**
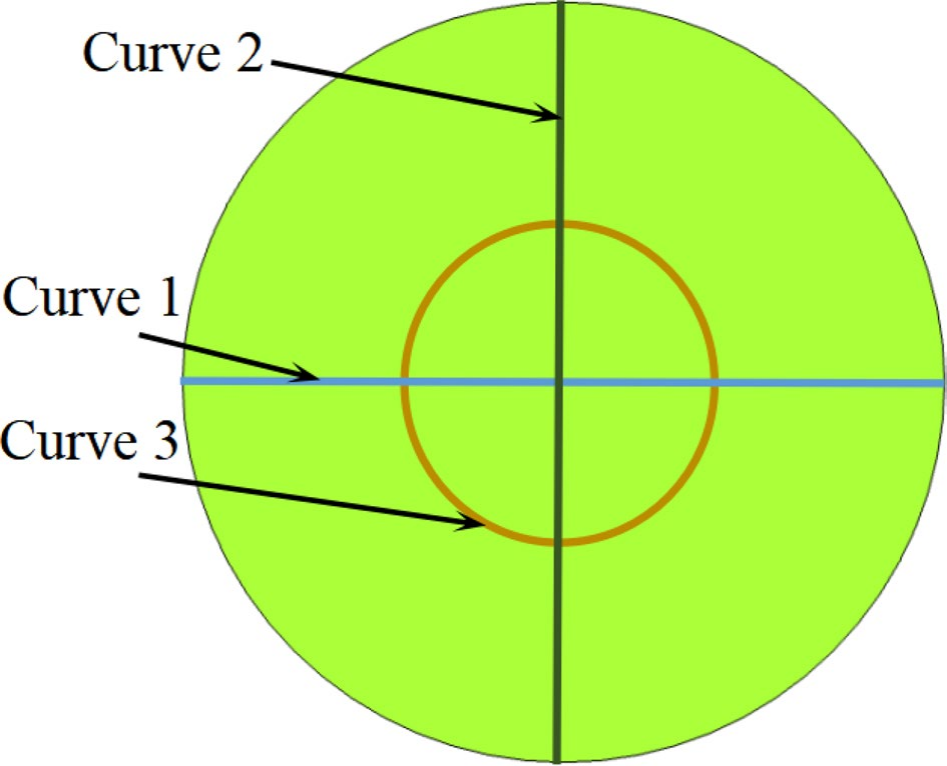
Curves used for far-field acoustic pressure calculation.

**Figure 21:**
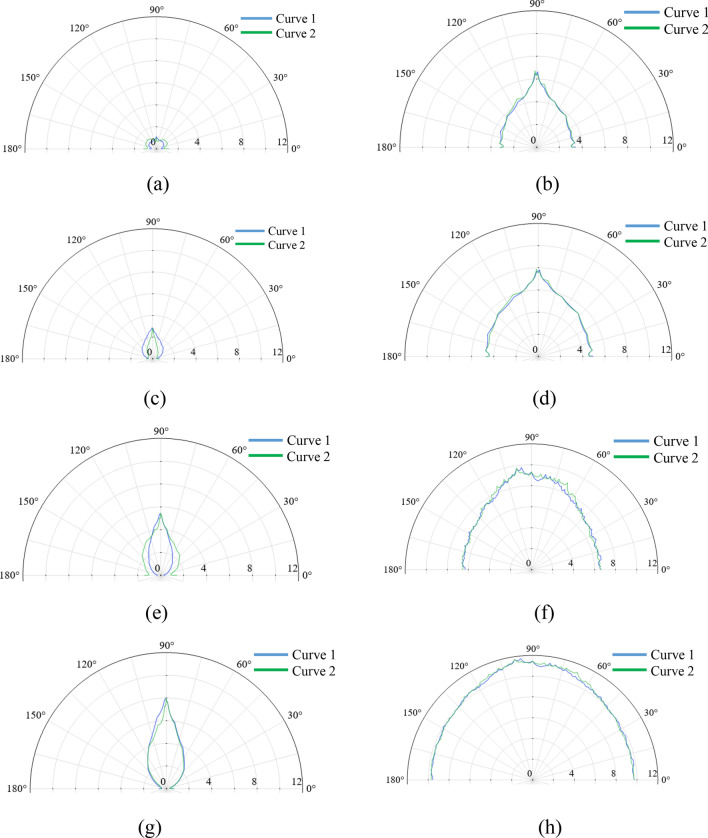
Polar diagram of the absolute value of far-field acoustic pressure (Pa) on curve 1 and 2 for different grazing flow Mach numbers. (**a**) PMM-ALD structure, $M_{x} = 0$. (**b**) Traditional ALD structure, $M_{x} = 0$. (**c**) PMM-ALD structure, $M_{x} = 0.05$. (**d**) Traditional ALD structure, $M_{x} = 0.05$. (**e**) PMM-ALD structure, $M_{x} = 0.1$. (**f**) Traditional ALD structure, $M_{x} = 0.1$. (**g**) PMM-ALD structure, $M_{x} = 0.15$. (**h**) Traditional ALD structure, $M_{x} = 0.15$.

**Figure 22:**
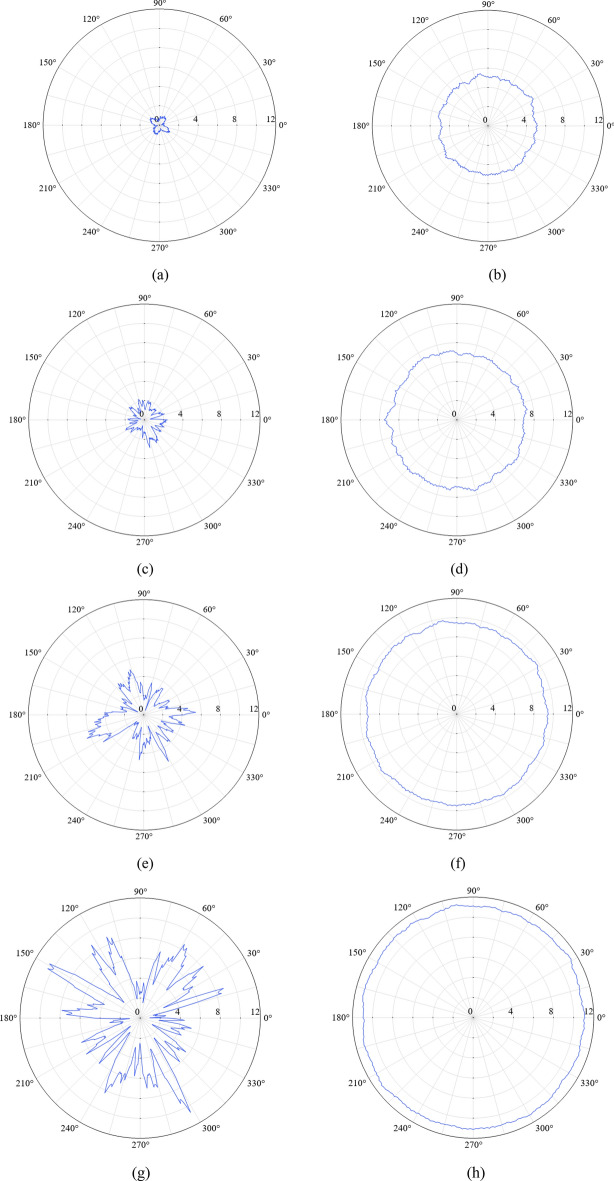
Polar diagram of the absolute value of far-field acoustic pressure (Pa) on curve 3 for different grazing flow Mach numbers. (**a**) PMM-ALD structure, $M_{x} = 0$. (**b**) Traditional ALD structure, $M_{x} = 0$. (**c**) PMM-ALD structure, $M_{x} = 0.05$. (**d**) Traditional ALD structure, $M_{x} = 0.05$. (**e**) PMM-ALD structure, $M_{x} = 0.1$. (**f**) Traditional ALD structure, $M_{x} = 0.1$. (**g**) PMM-ALD structure, $M_{x} = 0.15$. (**h**) Traditional ALD structure, $M_{x} = 0.15$.

Three curves on the far-field hemisphere surface are chosen to calculate the far-field acoustic pressure, as shown in Fig. 20. Curves 1 and 2 represent the arcs that follow the maximum horizontal and vertical circumferences, respectively. Curve 3 located at the right end of the hemisphere represent a circle with an area equal to that of the duct. Figures 21 and 22 gives the calculated absolute value of acoustic pressure on curves 1–3 in differnet Mach numbers of grazing flows. These results clearly demonstrate the advantages of the PMM-ALD structure in noise-reduction. Besides, for the transmission loss from the duct outlet to the circle area enclosed by curve 3, the PMM-ALD structure generates a transmission loss of 7.5 dB in the absence of grazing flow at 3800 Hz, while the ALD structure is 3.9 dB. The total transmission loss from the entrance of the structure to the far-field hemispherical surface is 32.5 dB for the PMM-ALD structure and 17.5 dB for the traditional ALD structure. Obviously, the spinning acoustic wave produced by the developed PMM can greatly enhance the noise-reduction performance of the traditional ALD with small length-diameter ratio.

## Conclusion

In order to enhance the noise-reduction performance of the ALD with small aspect ratios, a solution based on the incident wave phase-modulating was proposed. The basic idea is to transform the incidence plane wave into a spinning one by using the optimized PMM, and then use ALD for noise-reduction purpose. The simulation results demonstrated that the optimized PMM structure achieves an expected gradient phase distribution and successfully manipulate an incident plane wave into a spinning wave in circumferential mode, so that the noise-reduction performance of ALD can be greatly improved. By comparing with the COMSOL results, the effectiveness of the theoretical formulae for predicting phase shift and transmission losses has been demonstrated. Compared with the traditional ALD structure, the designed PMM- ALD structure exhibits excellent noise-reduction performance in the frequency ranges from 3300 to 4300 Hz in the presence of grazing flow. In addition, the far-field acoustic pressure also significantly decreases. This study provides a new approach for narrow band noise-reduction in a duct with relatively small length-diameter ratio.

## Data Availability

The datasets used and/or analysed during the current study available from the corresponding author on reasonable request.

## List of symbols

$a_{1} ,a_{2}$: Length of the backing cavity for PMM and ALD section, respectively
$A_{1} ,A_{2}$: Length of the fundamental PMM and ALD, respectively
$A_{m,n}$: Amplitude of the $(m,n)$ mode
AL: Acoustic liner
ALD: Acoustic liner duct
$b_{1} ,b_{2}$: Width of the backing cavity for PMM and ALD section, respectively
$B_{1} ,B_{2}$: Width of the fundamental PMM and ALD section, respectively
$c_{0}$: Acoustic velocity in air
$d_{1} ,d_{2}$: Diameter of the hole on the perforated plate for PMM and ALD section, respectively
$D_{1}$: Distance between the inlet and the first hole for PMM section
$D_{2}$: Distance between two adjacent holes for PMM section
$D_{3}$: Distance between the inlet of the duct and the ALD section
$g_{m,n}$: Cut-off ratio of $(m,n)$ mode
$h_{1} ,h_{2}$: Height of the hole on the perforated plate for PMM and ALD section, respectively
$h_{a}$: Corrected height of the hole on the perforated plate for PMM section
$J_{m}$: *m*Th order Bessel function
$$k$$: Wave number
$k_{1} ,k_{2}$: Axial wave number for the rigid wall and ALD section, respectively
$k_{x}$: Axial component of wave number
$\kappa_{1} ,\kappa_{2}$: Circumferential wave number for the rigid wall and ALD section, respectively
$l_{1} ,l_{2}$: Height of backing cavity for PMM and ALD section, respectively
$L_{1}$: Height of fundamental PMM
$$m$$: Circumferential mode
$M_{x}$: Mach number of grazing flow
$$n$$: Radial mode
${\mathbf{N}}_{1}$: Transfer matrix between the inlet and the first hole for PMM section
${\mathbf{N}}_{2}$: Transfer matrix between adjacent holes for PMM section
$$p$$: Acoustic pressure
$p_{1}$: Acoustic pressure from the inlet to the first hole for PMM section
$p_{i} \, (i \ge 2)$: Acoustic pressure between the $(i - 1){\text{th}}$ and the $i{\text{th}}$ holes for PMM section
$P_{i}^{ \pm }$: Propagation coefficient in the directions of $\pm x$ in the $i{\text{th}}$ hole for PMM section
PMM: Phase-modulating metasurface
$$R$$: Duct radius
$S_{e}$: Equivalent area of the duct for PMM section
$S_{h}$: Section area of the hole for PMM section
$t_{ij}$: Corresponding element $(i,j)$ in ${\mathbf{T}}$
$T$: Transmission coefficient of PMM
$${\text{TL}}$$: Transmission loss
${\mathbf{T}}_{1}$: Transfer matrix from the inlet to the first hole for PMM section
${\mathbf{T}}_{i} \, (i \ge 2)$: Transfer matrix between the $(i - 1){\text{th}}$ and the $i{\text{th}}$ holes for PMM section
$U_{1}$: Volume velocity from the inlet to the first hole for PMM section
$U_{i} \, (i \ge 2)$: Volume velocity between the $(i - 1){\text{th}}$ and the $i{\text{th}}$ holes for PMM section
$V_{2}$: Volume of the cavity of ALD section
$$W$$: Acoustic power
$Z_{c}$: Acoustic impedance of the cavity for PMM section
$Z_{d}$: Acoustic impedance in duct above the cavity for PMM section
$Z_{h}$: Acoustic impedance of the hole for PMM section
$Z_{l}$: Specific acoustic impedance of ALD
$Z_{t}$: Impedance in duct for PMM section
$$\alpha$$: Correction factor
$$\lambda$$: Acoustic wavelength
$$\sigma$$: Perforation rate
$\mu$: Dynamic viscosity of air
$\rho_{0}$: Density of air
